# Proteome Changes Induced by Imatinib and Novel Imatinib Derivatives in K562 Human Chronic Myeloid Leukemia Cells

**DOI:** 10.3390/proteomes2030363

**Published:** 2014-07-22

**Authors:** Katerina Arvaniti, Anastasia Papadioti, Maria Kinigopoulou, Vassiliki Theodorou, Konstantinos Skobridis, Georgios Tsiotis

**Affiliations:** 1 Division of Biochemistry, Department of Chemistry, University of Crete, P.O. Box 2208, GR-71003 Voutes, Greece; E-Mails: katarv87@gmail.com (K.A.); anastasia_papadioti@hotmail.com (A.P.); 2 Department of Chemistry, Section of Organic Chemistry and Biochemistry, University of Ioannina, 45110 Ioannina, Greece; E-Mails: m_kinigopoulou@hotmail.com (M.K.); vtheodor@cc.uoi.gr (V.T.); kskobrid@cc.uoi.gr (K.S.)

**Keywords:** kinase inhibitors, SILAC, protein kinases, imatinib, chronic myeloid leukemia

## Abstract

Imatinib mesylate is the leading compound to treat chronic myeloid leukemia (CML) and other cancers, through its inhibition of Bcr-Abl tyrosine kinases. However, resistance to imatinib develops frequently, particularly in late-stage disease and has necessitated the development of new Bcr-Abl inhibitors. The synthesis of a new series of phenylaminopyrimidines, structurally related to imatinib, showed large interest since the introduction of nilotinib. Here, we compare the protein levels in K562 cells treated with either imatinib or with novel imatinib derivates. Our results revealed that among the 986 quantified proteins, 35 had significantly altered levels of expression by imatinib or its derivates. In a second series of experiments, we directly compared the proteomes of imatinib treated K562 cells with those K562 cells treated with any of the four imatinib derivates. More than 1029 protein were quantified, 80 of which had altered levels of expression. Both experiments pointed to changes in the expression of the ATP-dependent RNA helicase DDX3X and of two mitochondrial coiled-coil-helix-coiled-coil-helix domain-containing proteins.

## 1. Introduction

Chronic myeloid leukemia (CML) is a disorder of the hematopoietic stem cells. The disorder arises from a translocation between regions of chromosomes 9 and 22 [1]. The translocation generates the Bcr-Abl fusion gene, which contains the *Bcr* gene of chromosome 22 and the *Abl* gene of chromosome 9 [1]. This gene encodes the Bcr-Abl tyrosine kinase and deregulated activity of this gene drives several proliferative and antiapoptotic pathways [2,3].

Imatinib (also known as imatinib mesylate or Glivec) was one of the first small molecule inhibitors developed for the targeted inactivation of kinases in human cancer. Imatinib inhibits the kinase activity of Bcr-Abl by direct association with the ATP-binding site. Imatinib treatment dephosphorylates the Bcr-Abl protein leading to its inactivation. The cellular effect is the interruption of the constitutive activation of signaling cascades, triggering apoptosis and arresting cell cycle progression [4]. However, CML patients in advanced disease states such as the accelerated and blast crisis phases, typically relapse and acquire resistance to imatinib within several months [5]. In the majority of these cases, resistance is due to mutations in the kinase domain encoding region of the Bcr-Abl oncogene, which selectively interfere with imatinib binding without abrogating the catalytic activity of the Abl tyrosine kinase [5,6,7].

Structural data revealed that imatinib interacts with an inactive conformation of the Abl kinase, which is destabilized by many imatinib resistance-conferring mutations [8]. These mechanistic insights provide a rational basis for the development of second-generation inhibitors [9,10,11], such as the small molecule drugs bosutinib and dasatinib, which target the active kinase conformation and thereby overcome imatinib resistance in many Abl kinase variants [5,6,7]. Recently, Skorbidis *et al.* have reported on the synthesis of a new series of imatinib derivatives based on modifications of the phenyl and N-methylpiperazine rings [12]. Preliminary results show that in general, these have a greater activity against the family of PDGF receptors and poorer activity against Abl [12]. However, our understanding of how changes in drug design impact biological activity is far from complete [13]. 

There have been studies on imatinib-induced perturbation in global protein expression in which 2-dimensional gel electrophoresis (2-DE) coupled with tandem mass spectrometry (MS/MS) was employed for protein identification and quantification [14,15,16]. Further, similar studies were performed to identify proteins likely involved in obtaining resistance against imatinib [15,16,17,18,19,20,21] Other than 2-DE, several stable isotope-labeling strategies, especially stable isotope labeling by amino acids in cell culture (SILAC) [22], have been developed for MS-based differential protein expression analysis. The SILAC approach is very efficient in quantifying a larger part of the proteome and in this context, SILAC was used together with LC-MS/MS to examine the proteome effect of imatinib-induced alterations of the Bcr-Abl kinase in CML cells [23].

Protein kinases are critical components of cellular signal transduction cascades by phosphorylation of their targets and represent an important class of drug target in the field of cancer [24]. Although phosphorylation is a critical step in cellular signaling, the dynamics of protein levels also need to be ascertained to accurately and fully comprehend the roles that kinase inhibitors play in the proteome responses. 

In the present study, we employed LC-MS/MS, along with differential SILAC labeling to assess alterations in protein levels in Bcr-Abl positive human K562 cells treated with imatinib and several imatinib derivatives. We quantified in these experiments more than 1000 proteins, among which 117 were found to be significantly altered. Furthermore, our study tries to understand the influence of the drug structure in its selectivity of protein expression perturbation.

## 2. Experimental

### 2.1. Materials

**Figure 1:**
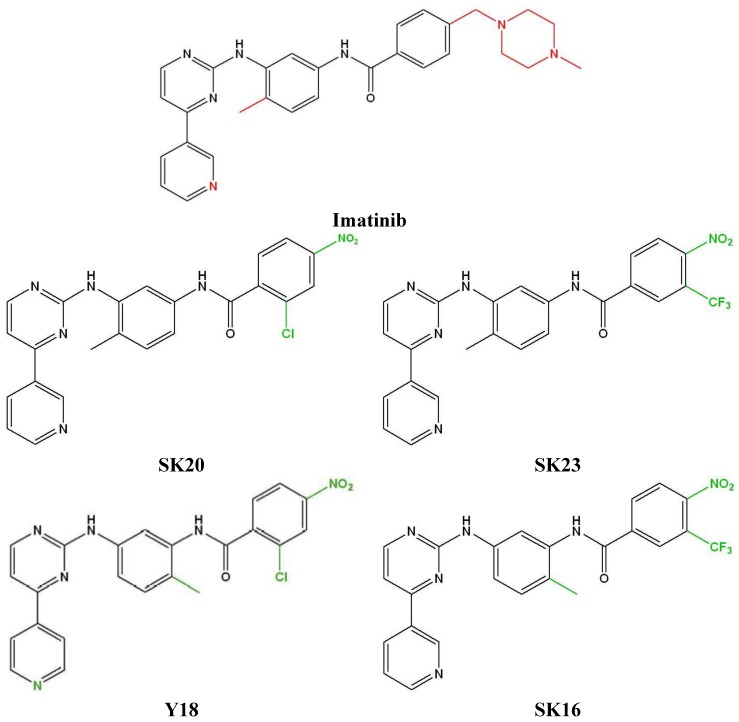
Structure of imatinib and its derivatives. Imatinib: N-(4-methyl-3-((4-(pyridin-3-yl)pyrimidin-2-yl)amino)phenyl)-4-((4-methylpiperazin-1 yl)methyl)benzamide; SK20: 2-chloro-N-(4-methyl-3-((4-(pyridin-3-yl)pyrimidin-2-yl)amino)phenyl)-4-nitrobenzamide; SK23: N-(4-methyl-3-((4-(pyridin-3-yl)pyrimidin-2-yl)amino)phenyl)-4-nitro-3-(trifluoro-methyl)benzamide; Y18: 2-chloro-N-(2-methyl-5-((4-(pyridin-4-yl)pyrimidin-2-yl)amino) phenyl)-4-nitrobenzamide; SK16: N-(2-methyl-5-((4-(pyridin-3-yl)pyrimidin-2-yl)amino) phenyl)-4-nitro-3-(trifluoromethyl)benzamide. Red color indicate atoms or groups which have been removed. Green color indicate the modifications compare to imatinib.

Imatinib as imatinib mesylate was a kind gift from Novartis Pharma AG (Basel, Switzerland). Synthesis and characterisation of the imatinib derivates SK16, 20 and 23 has been described previously (Figure 1) [12], whereas the synthesis and characterization of the Y18 compound is described in the Supplementary Data. Heavy lysine and arginine ([13C6]-L-lysine and [13C6]-L-arginine) were purchased from Silantes (München, Germany). RPMI 1640 medium for cell culture was purchased from Invitrogen (Paisley, Scotland, UK) and Silantes. Fetal calf serum, penicillin, streptomycin, PBS and glutamax were purchased from Gibco (Invitrogen). Tris(2-carboxyethyl)phosphine hydrochloride (TCEP-HCl) was purchased from Thermo Scientific (Boston, MA, USA). Modified porcine trypsin sequencing-grade was from Promega (Madison, WI, USA). 

### 2.2. Cell Culture

K562 cells (ATCC, American Type Culture Collection, Manassas, VA, USA, CCL-243) were cultured in RPMI medium supplemented with 10% fetal calf serum, penicillin (100 units/mL) and streptomycin (100 µg/mL). Cells were maintained in a humidified atmosphere with 5% CO_2_ at 37 °C, with medium renewal every 2–3 days.

### 2.3. SILAC Medium

For SILAC experiments, the RPMI medium without L-lysine or L-arginine was custom-prepared. The complete light and heavy RPMI media were prepared by the addition of light or heavy lysine and arginine at respective concentrations of 275 µM and 57.5 µM (*i.e.*, 5% of the suggested concentration present in RPMI at which L-arginine to proline conversion was not detectable), together with FBS and glutamax (5 mL/500 mL of medium).

### 2.4. Drug Treatment

After seven cell doublings, K562 cells grown in light medium at a density of approximately 4 × 10^5^ cells/mL were treated with 1 μM imatinib or an imatinib derivate for 24 h. The cells cultured in heavy medium, also after seven cell doublings, were treated with water in the case of imatinib or DMSO (0.001% final concentration) in the case of the derivates, respectively. Following drug treatment, the light and heavy cells were collected by centrifugation at 1250 *g* for 5 min and washed three times with ice-cold PBS. The cell pellet was re-suspended in 20 mM NH_4_CO_3_, pH 7.9 and lysed by freeze-thawing (three cycles). Cell lysates were centrifuged at 13,000 *g* at 4 °C for 30 min, and the resulting supernatants were collected. The protein concentration of the cell lysates was determined using the Bradford assay. 

### 2.5. Proteome Analysis

Equal amounts of heavy and light lysates were mixed (500 μg total). Guanidinium hydrochloride and acetonitrile were added to final concentrations of 0.5 M and 2%, respectively. Proteins were digested with trypsin at a 1:50 (w/w) enzyme/protein ratio, and digestion was allowed to perform overnight at 37 °C. Digestion was ended by adding 4 μL of acetic acid per 100 μL of sample. The resulting peptide mixtures were centrifuged at 13,000 *g* for 10 min and the supernatants were dried under vacuum in a centrifuge and re-dissolved in 20 μL of 2 mM tris(2-carboxyehyl)phosphine in 2% acetonitrile. For each set-up, two biological repeats were prepared, and their resulting digests were each time analyzed in triplicate (three technical replicates) by LC-MS/MS on an Ultimate 3000 HPLC system (Dionex, Amsterdam, The Netherlands) in-line connected to an LTQ Orbitrap XL mass spectrometer (Thermo Electron, Bremen, Germany). Here, a 180 min gradient from 2% acetonitrile to 35% acetonitrile, both in 0.1% formic acid, followed by a washing and re-equilibration step on an in-house packed 30 cm long column (75 µm inner diameter, Reprosil-Pur Basic C18-HD 3 µm, Dr. Maisch, Ammerbuch-Entringen, Germany) was used to elute the peptides from the trapping column (100 µm inner diameter, Reprosil-Pur Basic C18-HD 3 µm, Dr. Maisch). Instrument settings for LC-MS/MS analysis and the generation of MS/MS peak lists were used as previously described [25]. The Orbitrap XL mass spectrometer was operated in data-dependent mode, automatically switching between MS and MS/MS acquisition for the five most abundant peaks in a given MS spectrum. Full scan MS spectra were acquired in the Orbitrap at a target value of 1e6 with a resolution of 60,000. The six most intense ions were then isolated for fragmentation in the linear ion trap, with a dynamic exclusion of 60 s. Peptides were fragmented after filling the ion trap at a target value of 1e4 ion counts. From the MS/MS data in each LC run, Mascot Generic Files were created using the Mascot Distiller software (version 2.3.01, Matrix Science, London, UK). While generating these peak lists, grouping of spectra was allowed with a maximum intermediate retention time of 30 s and a maximum intermediate scan count of 5 was used where possible. Grouping was done with 0.005 Da precursor tolerance. A peak list was only generated when the MS/MS spectrum contained more than 10 peaks. There was no deisotoping and the relative signal to noise limit was set at 2. These peak lists were then searched with the Mascot search engine (Matrix Science) using the Mascot Daemon interface (version 2.3, Matrix Science). The spectra were searched in the human subsection of the Swiss-Prot database, release 2011_11 (20,251 entries). 

Methionine oxidation to methionine-sulfoxide, acetylation of the peptide N-terminus and pyroglutamate formation of N-terminal glutamine were set as variable modifications. SILAC labeling of lysine and arginine (+6 Da) was used for quantitation. The protease setting was on trypsin with one missed cleavage allowed. The mass tolerance on the precursor ion was set to ±10 ppm and on fragment ions to ±0.5 Da. In addition, Mascot’s C13 setting was set to 1. Only peptides that were ranked one and had an ion score at least equal to the corresponding identity threshold at 99% confidence were withheld and further data handling was done in the ms_lims database [26]. Peptide quantifications were carried out using the Mascot Distiller Quantitation Toolbox (version 2.2.1). The quantification method details were as follows: constrain search, yes; protein ratio type, average; report detail, yes; minimum peptides, 1; protocol, precursor; allow mass time match, yes; allow elution shift, no; all charge states, yes; correlation threshold, 0.97; standard error threshold, 0.16; fraction threshold, not ticked. The mean was used to calculate the protein ratio, number of peptides: 1. For further analysis, proteins identified by only a single peptide were omitted. Ratios for identified proteins were calculated by comparing the XIC peak areas of all matched light peptides with those of the heavy peptides, and the results were verified by visual inspection of MS spectra with the Rover tool [27].The false discovery rate (FDR) values on the data from the individual analyses were calculated by dividing the hits in the reversed database by the positive hits in the forward database [28]. A reversed version of the human Swiss-Prot database was made by using DBToolkit [29], and peak lists were also searched in this database. The results of the forward and of the reversed searches were previewed in ms_lims. All identified MS/MS spectra are publicly available in the PRIDE database under the experiment number PXD000216 [30,31]. Analysis of biological pathways involved was done using Pathway Commons, a web resource for biological pathway data [32]. 

## 3. Results and Discussion

Two potent second-generation BCR-ABL inhibitors have been recently developed, nilotinib and dasatinib. In the case of nilotinib, the highly polar and basic N-methylpiperazine heterocycle of imatinib was replaced by alternative binding groups to generate nilotinib, which is a greatly improved inhibitor of Abl, and is also effective against most cases of imatinib resistance caused by point mutations of Abl [33,34]. Based on this concept, alternations in the phenyl and N-methylpiperazine rings were introduced [12]. 

To gain insight into the molecular pathways perturbed by imatinib or imatinib derivatives, we used SILAC in combination with LC-MS/MS to assess possible changes in protein expression induced by these compounds in K562 cells. The investigated compounds have IC_50_ values in the range of 0.1–1 μM and, in order to have comparable results, we chose a dose of 1 μM for imatinib and their derivates [21,35]. Trypan blue exclusion assays showed less than 5% cell death at this dose after a 24 h treatment [21]. To obtain reliable results, we carried out the SILAC experiments in three technical replicates from each biological repeat (two biological repeats were analyzed per set-up) (Table 1). 

**Table 1:** Synopsis of the protein identification results of the SILAC experiments.

Study	Biological Sample	Spectra		Identifications FDR		Unique Peptides		Unique Proteins		Common Proteins
		1	2	1	2	1	2	1	2	1+2
K562 water/K562 DMSO		56,140	-	13,584 0.08%		3437		902		
K562/K562 Imatinib		51,261	62,478	11,138 0.116%	13,740 0.116%	3530	3636	834	874	798
K562/K562 SK23		56,763	63,385	13,524 0.064%	13,733 0.094%	3625	3456	878	844	697
K562/K562 SK20		50,833	65,130	12,882 0.085%	14,682 0.108%	3286	3703	831	854	671
K562/K562 SK16		53,993	64,528	13,433 0.089%	14,095 0.127%	3277	3528	841	858	676
K562/K562 Y18		50,399	64,564	12,593 0.103%	14,759 0.074%	3084	3656	796	855	645
K562 Imatinib/ K562 SK23		56,398	50,906	14,958 0.06%	11,105 0.098%	4345	3168	980	846	846
K562 Imatinib/ K562 SK20		53,377	52,531	14,376 0.097%	12,456 0.104%	4167	3497	912	848	834
K562 Imatinib/ K562 SK16		58,970	52,426	15,278 0.071%	12,172 0.073%	4167	3530	934	868	852
K562 Imatinib/ K562 Y18		58,311	53,522	15,410 0.038%	11,584 0.103%	4186	3442	922	865	853

**Figure 2:**
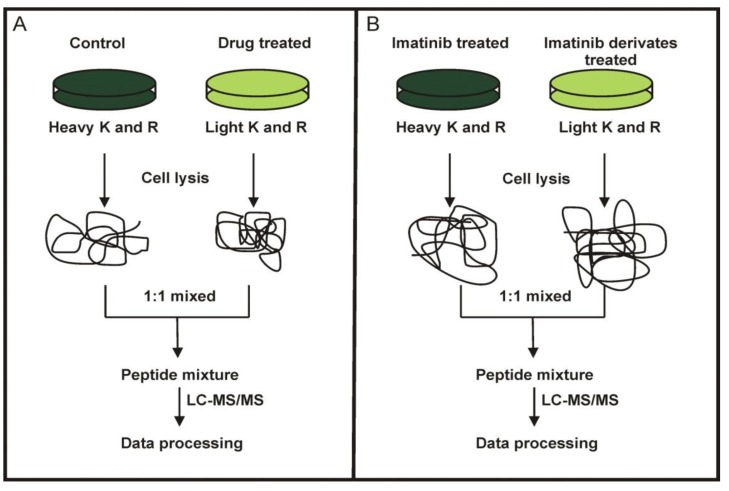
SILAC labeling workflow combined with LC-MS/MS for the comparative analysis of protein expression in K562 cells upon treatment with imatinib and its derivatives (**A**) and direct comparison of the proteomes of K562 cells treated with imatinib or its derivates (**B**).

In the first set of SILAC experiments, K562 cells were used to compare the effects of imatinib with the new imatinib derivates (Figure 2A). In the second set of SILAC experiments, we directly compared the proteomes of imatinib treated K562 cells with those K562 cells treated with any of the four imatinib derivates (Figure 2B). 

### 3.1. SILAC Proteome Analyses of K562 Cells Treated with Imatinib or Imatinib Derivatives

Until now, only one study [21] has used SILAC coupled with LC-MS/MS to identify and quantify proteome changes in K562 cells treated with imatinib or imatinib derivates. Here, we harvested the proteomes from K562 cells and drug-treated K562 cells and compared protein expression differences. In the first set of experiments, we were able to quantify a total of 986 proteins (Table 2 and Table S1, Supplementary Material). Peptides with a ratio outside of the 95% confidence interval of the population distribution were noted as significantly altered. In order to identify such altered proteins, they should be quantified in both biological samples. Together, 35 proteins were found significantly altered upon imatinib or imatinib derivates treatment (Table 2). Furthermore, the distribution of the identified proteins among cellular compartments was determined. Annotated proteins belong to eight cellular compartments (Table 2). While imatinib affected the expression level of only seven proteins, three of its derivates changed the expression level of 11–24 proteins, except for compound SK16 which affected the expression of four proteins only. 

**Table 2:** Altered protein expression between in K562 cells after treatment with imatinib or its derivates. +, indicates identified proteins. −, indicates proteins that have not been identified. ↓ and ↑ indicate proteins that are down or up-regulated, respectively, upon inhibitor treatment.

Accession	Protein Name	Localization	SK23	SK16	SK20	Y18	Imatinib
**Amino acid biosynthesis**							
O43175	D-3-phosphoglycerate dehydrogenase	cytoplasm	+	+	+	↑	+
**Apoptosis-cell cycle**							
P61289	Proteasome activator complex subunit 3	nucleus	−	−	−	↓	−
Q00526	Cyclin-dependent kinase 3	cytoplasm	↑	−	+	+	−
Q92688	Acidic leucine-rich nuclear phosphoprotein 32 family member B	cytoplasm	↓	+	+	+	+
**DNA and RNA related proteins**							
Q16630	Cleavage and polyadenylation specificity factor subunit 6	nucleus	+	+	+	↓	+
P22087	rRNA 2'-O-methyltransferase fibrillarin	nucleus	+	+	+	↓	+
Q9NR30	Nucleolar RNA helicase 2	nucleus	↑	+	+	↓	+
Q08211	ATP-dependent RNA helicase A	cytoplasm	+	+	+	↓	+
**Other**							
P62714	Serine/threonine-protein phosphatase 2A catalytic subunit beta isoform	centromere	↓	+	+	+	+
P09622	Dihydrolipoyl dehydrogenase,	mitochondrion	+	+	↑	↑	+
Q96EP5	DAZ-associated protein 1	cytoplasm	+	+	+	↓	+
P13646	Keratin, type I cytoskeletal 13	intermediate filament	↓	−	−	−	−
P49327	Fatty acid synthase	cytoplasm	+	+	↓	↓	+
P07237	Protein disulfide-isomerase	cell memb.	↑	+	↑	↑	+
P49915	GMP synthase [glutamine-hydrolyzing]	cytoplasm	+	+	+	↓	+
**Host-virus interaction**							
O00571	ATP-dependent RNA helicase DDX3X	cytoplasm	↑	↑	↑	↑	↑
Q13435	Splicing factor 3B subunit 2	nucleus	↑	+	+	+	+
**Protein biosynthesis**							
Q15084	Protein disulfide-isomerase A6	cell memb.	+	+	↑	+	+
P41567	Eukaryotic translation initiation factor 1	cytoplasm	↑	+	↑	↑	+
O60739	Eukaryotic translation initiation factor 1b	unknown	↓	+	↓	+	+
**Signal transduction**							
P49006	MARCKS-related protein	cell memb.	↑	+	+	+	↑
P30101	Protein disulfide-isomerase A3	en reticulum	+	+	↑	+	+
**Stress response**							
Q14011	Cold-inducible RNA-binding protein	cytoplasm	+	↑	+	↑	+
P98179	Putative RNA-binding protein 3	cytoplasm	−	↑	−	↑	−
Q9Y4L1	Hypoxia up-regulated protein 1	en reticulum	+	+	↑	↑	+
**Transcription**							
P43243	Matrin-3	nucleus	+	+	+	↓	+
Q96AE4	Far upstream element-binding protein 1	nucleus	+	+	+	↓	+
**Transport**							
Q9UN86	Ras GTPase-activating protein-binding protein 2	cytoplasm	−	↓	+	−	−
**Unknown**							
Q5T1J5	Putative coiled-coil-helix-coiled-coil-helix domain-containing protein CHCHD2P9,	mitochondrion	↓	↓	↓	↓	↓
Q9Y6H1	Coiled-coil-helix-coiled-coil-helix domain-containing protein 2	mitochondrion	↓	↓	↓	↓	↓
Q9BQ39	ATP-dependent RNA helicase DDX50	nucleus	↑	+	+	↓	+
Q13067	G antigen 3	unknown	+	+	+	↓	↓
Q13069	G antigen 5	unknown	+	+	+	↓	↓
Q13070	G antigen 6	unknown	+	+	+	↓	↓

From the 35 altered proteins, an ATP-dependent RNA helicase DDX3X (O00571) was found to be up-regulated after treatment with imatinib or imatinib derivates. This protein has been implicated to play a role in several processes regulating gene expression, including transcription, splicing, mRNA export and translation. It has also been suggested to be involved in cell cycle control and the regulation of apoptosis. In addition, DDX3X was recently shown to be part of innate immune signaling pathways and to contribute to the induction of anti-viral mediators, such as type I interferon [36]. DDX3X interacts with a variety of proteins, and most of them are eukaryotic translation initiation factors and, for some of these proteins, these interactions are regulated by phosphorylation. A second protein which was up-regulated after treatment with imatinib (and SK23) is a MARCKS related protein (P49006) which might be involved in coupling protein kinase C and calmodulin signal transduction systems. 

Imatinib induces down-regulation of two mitochondrial coiled-coil-helix-coiled-coil-helix domain-containing proteins (Q5T1J5, Q9Y6H1) which are in fact down-regulated by all derivatives. Such proteins have been reported to be required for the maintenance of mitochondrial crista integrity and mitochondrial function [37,38], by acting as scaffolding proteins that stabilize protein complexes involved in crista architecture and protein import [38]. Mitochondria play an important role in energy metabolism and apoptosis, underline the importance of this protein for the cells’ energy metabolism [39,40]. 

In addition, three proteins belonging to the GAGE family proteins (Q13067, Q13069, Q13070) were down-regulated by imatinib and Y18. These proteins are tissue-specific and are expressed in a variety of tumor tissues but not in normal tissues [41]. Next to these proteins, a sulfide isomerase (P07237) and a eukaryotic translation initiation factor 1 (P41567), both involved in protein synthesis, were up-regulated by SK23, SK20 and Y18. Eleven proteins were found to alter their expression by only two drugs and 17 by only one drug (Table 2). These proteins are involved in different cellular function such as protein synthesis, stress response and amino acid biosynthesis (Table 2). Of note, in some cases the investigated drugs had opposite effects on the expression level of a given protein (SK23 up and Y18 down) as is the case for the nucleolar RNA helicase 2 (Q9NR30) and the ATP-dependent RNA helicase DDX50 (Q9BQ39) (Table 2). Both proteins belong to the DDX21/DDX50 subfamily of the DEAD box helicase family and contain multiple phosphorylation sites [42], indicating that the compounds had a reverse effect in the same pathway. 

The number of quantified proteins in our study is roughly about 75% of that reported in a recent SILAC study based on separation of K562 proteins by SDS-PAGE followed by LC-MS/MS analysis [21]. In that study, more than 70 proteins were found significantly altered upon imatinib exposure. These proteins are involved in erythroid differentiation and mRNA translation, and further include histones and several enzymes. Although most of the already reported proteins with altered expression following imatinib treatment were identified in our study, seven proteins with altered expression found here were not reported in the previous study. Variation imposed by differences in protein extraction, and their separation prior to mass spectrometry might account for the observed differences [14,16,21]. In contrast to our gel-free separation of the tryptic peptides generated form the whole proteome, the previous study applied gel based methods (1DE or 2DE) for the proteome separation. 

### 3.2. Direct Comparison of the Proteomes of K562 Cells Treated with Imatinib or Its Derivates

In order to further characterize the biological effects induced by the new drugs, we applied the SILAC strategy to K562 cells treated with imatinib compared with K562 cells treated with the new imatinib analogues (Figure 1). To obtain reliable data, we filtered all SILAC-based quantifications for proteins that were recorded in both biological replicate experiments as mentioned above. We applied the same protocol and the same concentration of the substances as in the first experiment. In total, we quantified more than 1100 proteins (Supplementary Table S2,). Protein ratios for imatinib treated K562 cells *versus* treated K562 cells with the imatinib analogues allowed the identification of 80 proteins with altered expression levels, and 46 proteins were up-regulated, while 34 were down-regulated (Table 3). These altered proteins belonged to eight cellular compartments. Most of them (30) are involved in nucleosome assembly, while the number of proteins involved in cell cycle regulation, protein biosynthesis, transcription and translation is much smaller. Most of the altered proteins were obtained in the comparison of imatinib *versus* Y18 (Table 3). 

**Table 3:** Altered Protein Expression between the K562 after treatment with imatinib and the K562 with imatinib derivates. +, indicates proteins that are present in the samples. − indicates proteins that have not been identified. ↓ and ↑ indicate proteins that are down or up-regulated respectively upon inhibitor treatment.

Accession	Protein Name	Localization	SK23	SK16	SK20	Y18
**Cell cycle**						
Q13501	Sequestosome-1	Cytoplasm	↓	↓	↓	↓
P60323	Nanos homolog 3	Cytoplasm	↑	−	−	−
Q92688	Acidic leucine-rich nuclear phosphoprotein 32 family member B	Cytoplasm	↓	+	+	+
**Host-virus interaction**						
O00571	ATP-dependent RNA helicase DDX3X	Cytoplasm	↑	+	+	↑
P28070	Proteasome subunit beta type 4	Cytoplasm	+	↑	+	+
P23497	Nuclear autoantigen Sp-100	Nucleus	−	↑	−	−
**DNA and RNA related**						
Q9UMS4	Pre-mRNA-processing factor 19	cytoplasm	+	+	+	↓
P33316	Deoxyuridine 5'-triphosphate nucleotidohydrolase	mitochondrion	+	+	↑	+
Q9NR30	Nucleolar RNA helicase 2	nucleus	+	+	+	↓
P63162	Small nuclear ribonucleoprotein-associated protein N	nucleus	+	+	↓	+
P22087	rRNA 2'-O-methyltransferase fibrillarin	nucleus	+	+	+	↓
Q8WW01	tRNA-splicing endonuclease subunit Sen15	nucleus	−	↓	−	+
P14678	Small nuclear ribonucleoprotein-associated proteins B and B'	nucleus	+	+	↓	+
**Nucleosome assembly**						
P62805	Histone H4	chromosome	+	↑	↑	↑
O60814	Histone H2B type 1-K	chromosome	+	+	+	↑
P06899	Histone H2B type 1-J	chromosome	+	+	+	↑
P23527	Histone H2B type 1-O	chromosome	+	+	+	↑
P57053	Histone H2B type F-S	chromosome	+	+	+	↑
P58876	Histone H2B type 1-D	chromosome	+	+	+	↑
P62807	Histone H2B type 1-C/E/F/G/I	chromosome	+	+	+	↑
Q16778	Histone H2B type 2-E	chromosome	+	+	+	↑
Q5QNW6	Histone H2B type 2-F	chromosome	+	+	+	↑
Q8N257	Histone H2B type 3-B	chromosome	+	+	+	↑
Q93079	Histone H2B type 1-H	chromosome	+	+	+	↑
Q96A08	Histone H2B type 1-A	chromosome	+	+	+	↑
Q99877	Histone H2B type 1-N	chromosome	+	+	+	↑
Q99879	Histone H2B type 1-M	chromosome	+	+	+	↑
Q99880	Histone H2B type 1-L	chromosome	+	+	+	↑
P84243	Histone H3.3	chromosome	+	−	−	↑
Q16695	Histone H3.1t	chromosome	+	−	−	↑
**Nucleosome assembly**						
Q6NXT2	Histone H3.3C	chromosome	+	−	−	↑
Q71DI3	Histone H3.2	chromosome	+	−	−	↑
P04908	Histone H2A type 1-B/E	nucleus	+	+	+	↑
P0C0S8	Histone H2A type 1	nucleus	+	+	+	↑
P20671	Histone H2A type 1-D	nucleus	+	+	+	↑
Q16777	Histone H2A type 2-C	nucleus	+	+	+	↑
Q6FI13	Histone H2A type 2-A	nucleus	+	+	+	↑
Q7L7L0	Histone H2A type 3	nucleus	+	+	+	↑
Q93077	Histone H2A type 1-C	nucleus	+	+	+	↑
Q96KK5	Histone H2A type 1-H	nucleus	+	+	+	↑
Q99878	Histone H2A type 1-J	nucleus	+	+	+	↑
Q9BTM1	Histone H2A.J	nucleus	+	+	+	↑
P68431	Histone H3.1	chromosome	+	−	−	↑
**Protein biosynthesis**						
Q15046	Lysine—tRNA ligase	cell membrane	↓	↓	↓	+
P41567	Eukaryotic translation initiation factor 1	cytoplasm	+	↓	+	+
O60739	Eukaryotic translation initiation factor 1b	unknown	+	↓	+	+
P07339	Cathepsin D	lysosome	+	+	+	↑
O00567	Nucleolar protein 56	cytoplasm	−	−	−	↓
Q14137	Ribosome biogenesis protein BOP1	nucleus	−	−	−	↓
O60884	DnaJ homolog subfamily	cell membrane	+	−	↓	−
**Other**						
P17516	Aldo-keto reductase family 1 member C4	cytoplasm	↑	↑	+	↑
Q9UKK9	ADP-sugar pyrophosphatase	intracellular	+	+	↑	↑
P52895	Aldo-keto reductase family 1 member C2	cytoplasm	+	↑	+	+
P02765	Alpha-2-HS-glycoprotein	secreted	↓	↓	↓	↓
Q09666	Neuroblast differentiation-associated protein AHNAK	nucleus	↑	↑	↑	↑
Q9UG63	ATP-binding cassette sub-family F member 2	mitochondrial envelope	−	−	−	↓
P25705	ATP synthase subunit alpha, mitochondrial	cell membrane	−	+	−	↑
P08758	Annexin A5	cytoplasm	+	+	↑	↑
P05937	Calbindin	cytoplasm	−	↑	−	+
P02768	Serum albumin	secreted	−	↓	−	−
P11171	Protein 4.1	cytoskeleton	↑	+	−	−
Q14011	Cold-inducible RNA-binding protein	cytoplasm	+	+	+	↑
P35908	Keratin, type II cytoskeletal 2 epidermal	intermediate filament/keratin	−	−	−	↓
**Transcription**						
Q01844	RNA-binding protein EWS	cell membrane	+	↑	+	+
Q96AE4	Far upstream element-binding protein 1	nucleus	+	+	+	↓
**Translation**						
Q16222	UDP-N-acetylhexosamine pyrophosphorylase	cytoplasm	↓	↓	−	↓
P05386	60S acidic ribosomal protein P1	cytoplasm	+	+	+	↓
P18621	60S ribosomal protein L17	cytoplasm	↓	+	+	+
Q04637	Eukaryotic translation initiation factor 4 gamma 1	cytoplasm	+	↓	+	+
Q9Y5S9	RNA-binding protein 8A	cytoplasm	+	+	+	↓
**unknown**						
O15523	ATP-dependent RNA helicase DDX3Y	cytoplasm	↑	−	−	+
Q5T1J5	Putative coiled-coil-helix-coiled-coil-helix domain-containing protein CHCHD2P9	mitochondrion	↓	↓	↓	↓
Q9Y6H1	Coiled-coil-helix-coiled-coil-helix domain-containing protein 2	mitochondrion	↓	↓	↓	↓
Q9BQ39	ATP-dependent RNA helicase DDX50	nucleus	+	+	+	↓
Q13067	G antigen 3	unknown	+	+	+	↓
Q13069	G antigen 5	unknown	+	+	+	↓
Q13070	G antigen 6	unknown	+	+	+	↓
Q8N7X1	RNA-binding motif protein, X-linked-like-3	unknown	−	−	↓	−

The common proteins, namely the sequestosome-1 (Q13501), two coiled-coil-helix-coiled-coil-helix domain-containing proteins (Q5T1J5, Q9Y6H1) and the alpha-2-HS-glycoprotein (P02765) were down-regulated, whereas a neuroblast differentiation-associated protein (Q09666) was up-regulated. In the first experiment, the sequestosome-1 protein, which is involved in cell differentiation, apoptosis, immune response and bone diseases [43,44,45], was not affected by the different drugs treatments. In this experiment however, our data showed down-regulation of this protein by the four imatinib derivatives as compared to imatinib. The coiled-coil-helix-coiled-coil-helix domain-containing proteins were found to be down-regulated by imanitib, and its level is further decreased by the four imatinib derivatives. The alpha-2-HS-glycoprotein promotes endocytosis, possesses opsonic properties and influences the mineral phase of bone. While the protein has been identified in the first experiment, it is observed to be further down-regulated by the new imatinib derivatives. Another interesting finding is the neuroblast differentiation-associated protein located in the nucleus and required for neuronal cell differentiation [46]. In the first experiment, this protein was identified only in K562 cells treated with imatinib in both biological repeats (Supplementary Table S1), whereas here it is found up-regulated by the imatinib derivatives compared to imatinib (Table 3). The protein contains more than 60 phosphorylation sites [42]. 

The proteins, with a change in their expression by three different treatments, were UDP-N-acetylhexosamine pyrophosphorylase (Q16222), lysine-tRNA ligase (Q15046), histone H4 (P62805), UDP-N-acetylhexosamine pyrophosphorylase (Q16222) and aldo-keto reductase (P17516) (up-regulated). Four proteins showed altered expression in two treatments; namely the ATP-dependent RNA helicase DDX3Y (O00571), annexin A5 (P08758), ADP-sugar pyrophosphatase (Q9UKK9) that were up-regulated and the S100-P protein (P25815) was down-regulated. The RNA helicase DDX3X was identified in all comparisons between imatinib and imatinib derivates. Further, SK23 and Y18 induce up-regulation of this protein indicating an increased expression of the ATP-dependent RNA helicase DDX3X compared to imatinib. Finally, 58 proteins were identified in only one treatment (with Y18) and they participate in nucleosome assembly (histones) or in the formation of intermediate filament. This is expected as judged from the two biological replicates of Y18 derivate where respectively 117 and 76 proteins that were found with altered levels. 

### 3.3. Structural Effects

Imatinib provides an excellent model system to investigate how changes in drug design impact biological activity. Recently, the synthesis of a new series of imatinib derivatives has been reported which show greater activity against the family of PDGF receptors and poorer activity against Abl, as a result of modifications of the phenyl and N-methylpiperazine rings [12]. Complementary to these studies, analysis of the proteome changes induced by these new compounds (Figure 1), showed that the expression of three proteins was changed in a way similar to imatinib. The ATP-dependent RNA helicase DDX3X (O00571) protein is part of the TNF alpha/NF-kB pathway, which is involved in the regulation of a wide spectrum of biological processes including cell proliferation, differentiation, apoptosis, lipid metabolism, and coagulation, while the two other coiled-coil-helix-coiled-coil-helix domain-containing proteins (Q5T1J5 Q9Y6H1) are not involved in cellular signaling. Replacing of the N-methylpiperazine ring with nitro and trifluoromethyl groups in the case of the SK23 compound (Figure 1) leads to a two fold increase in the number of affected proteins compared to imatinib. Three of them, a serine/threonine-protein phosphatase 2A (P62714), a disulfide-isomerase (P07237) and a splicing factor 3B (Q13435) are involved in different signaling pathways (FGFR, interferon, ALK1, SMAD2, cytokine signaling pathways), metabolism (lipoprotein and lipids) and mRNA splicing and processing. The importance of the position of the methyl group in the aromatic ring is demonstrated by the change of the o-methyl group (SK23) to a p-methyl group (SK16) as this reduced the number of affected proteins to only six, with only three common to imatinib treatment (Figure 1 and Table 2). Only one, the down regulated ras GTPase-activating protein-binding protein 2, is involved in the TNF alpha/NF-kB pathway as the ATP-dependent RNA helicase DDX3X (O00571). In addition, replacement of the trifluoromethyl (SK23) with a chloro group (SK20) affected 11 proteins, with four in common with SK23 and three with imatinib (Table 2). The up-regulated proteins take part in the metabolism of pyruvate (Dihydrolipoyl dehydrogenase, P09622), in lipid and lipoprotein metabolism (Protein disulfide-isomerase, P07237), in antigen processing pathways (Protein disulfide-isomerase A3, P30101) and in the unfolded protein response (Hypoxia up-regulated protein 1, Q9Y4L1), while the down-regulated fatty acid synthase is involved in fatty acid and triacylglycerol biosynthesis. A dramatic increase of the number of affected proteins was observed by changing the methyl group in the C ring and simultaneously replacing the 3 pyridyl ring to a 4 pyridyl ring (Y18) pointing out the importance of the interaction of the 3 pyridyl ring with the active site of the kinase. From the 24 affected proteins, nine were only affected by this compound (Table 2). These proteins participate in amino acid biosynthesis (D-3-phosphoglycerate dehydrogenase, O43175), in G1/S DNA damage checkpoints (Proteasome activator complex subunit 3, P61289), in the TNF alpha signaling pathway (rRNA 2'-O-methyltransferase fibrillarin, P22087), in RNA splicing and maturation (ATP-dependent RNA helicase A, Q08211) and in purine and nucleotide metabolism (GMP synthase, P49915).

## 4. Conclusions

Targeted intervention strategies with kinase inhibitors have already made an enormous impact on the treatment of several human cancers. Proteomics approach can contribute valuably with information to such efforts, including drug selectivity assessments in relevant biological systems. In this study, we have used proteomics to identify proteins whose levels are affected by imatinib or four of its new derivatives. Of note is that the number of identified and quantified proteins might have been higher if peptide pre-fractionation preceding LC-MS/MS analysis would have been included. Nevertheless, it was possible to gain insight on the cellular responses evoked by the analyzed drugs. Future studies on the cellular targets of these drugs may also facilitate the discovery of additional molecular pathways that are altered by imatinib and its derivates. This may contribute to an improved understanding of the induced cytotoxicity and the development of resistance towards the drugs. Taken together, the pharmacoproteomic profiling could constitute a valuable tool for the identification of drug-responsive biomarkers and for the establishment of a molecular basis for developing novel and more effective approaches for the therapeutic intervention of human CML.

Supplementary data to this article describe the synthesis and characterization of the Y18 compound and contain lists of identified and quantified proteins in all experiments.

## Supplementary Materials

Supplementary File 1
