# Supplementary material for: Proteome Changes Induced by Imatinib and Novel Imatinib Derivatives in K562 Human Chronic Myeloid Leukemia Cells
Source: Proteomes. 2014 Jul 22;2(3):363–81. doi: 10.3390/proteomes2030363 (PMC5302748; doi:10.3390/proteomes2030363)
Supplement: Supplementary File 1 [file proteomes-02-00363-s001.zip › proteomes-55891-supplementary-final.pdf]

# Supplementary Material for

# Proteome Changes Induced by Imatinib and Novel Imatinib Derivatives in K562 Human Chronic Myeloid Leukemia Cells

## Synthesis of compound Y18

2-chloro-N-(2-methyl-5-((4-(pyridin-4-yl)pyrimidin-2-yl)amino)phenyl)-4 nitrobenzamide

### General Methods and Materials

Solvents used were *puriss*, except for THF which was purified by fresh distillation over sodium/benzophenone. Commercially available reagents were used as received, without any further purification. Analytical TLC was performed on commercial Merck silica gel 60 $F_{254}$. Flash chromatography was carried out using silica gel 60 (230–400 mesh). The melting points (uncorrected) were determined with a Buchi 510 apparatus. IR spectra were recorded on a Perkin-Elmer FT-IR 1600 spectrometer. $^{1}$H-NMR spectra were recorded on a Bruker AMX (400 MHz and 250 MHz) spectrometer using tetramethylsilane (TMS) as internal standard and chemical shifts are reported in ppm (δ) referenced to TMS, and coupling constants *J* in Hz. The high-resolution ESI mass spectrum of the final compound **Y18** was obtained using a ThermoFisher Scientific Orbitrap XL spectrometer.

### General Description of the Synthesis of the Final Compound Y18

The target compound **Y18** was prepared according to the previously described procedure for the synthesis of pyrimidine ring systems [1–4] as shown in Scheme S1. Thus, 4-acetyl pyridine **1** using *N,N*-dimethylformamide-diethylacetal was converted into the corresponding enaminones **2** which were then let to react with 4-methyl-3-nitrophenylguanidine hydrochloride (substituted phenylguanidine hydrochloride of the aniline derivative 4-methyl-3-nitroaniline) to give the phenylamino-pyrimidine **3**. Catalytic hydrogenolysis of the nitro group with $PtO_2$ (*Adam's* catalyst) was then performed to produce the corresponding aniline **4** which was subsequently coupled with 2-chloro-4-nitrobenzoyl chloride to give the final compound **Y18**.

**Scheme S1.** Synthetic route of the inhibitor Y18. Reagents and conditions: (**a**) *N*,*N*-dimethylformamide diethylacetal, *o*-xylene, reflux; (**b**) HCl, cyanamide; (**c**) *i*propanol, $Na_2CO_3$, reflux; (**d**) $PtO_2$, $H_2$, THF, room temperature; (**e**) triethylamine, 2-chloro-4-nitrobenzoyl chloride, THF, room temperature.

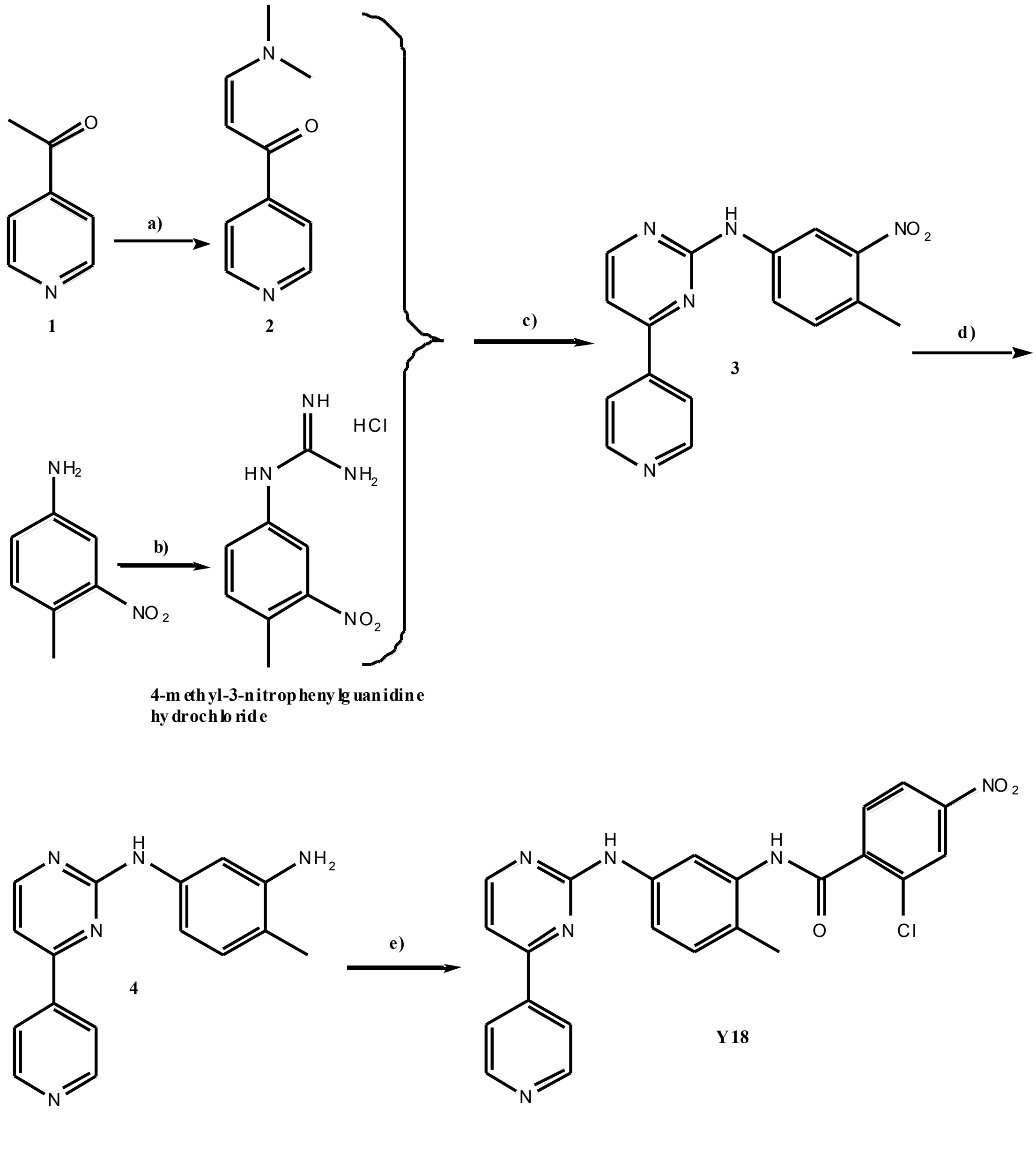


**3-dimethylamino-1-(4-pyridyl)-2-propen-1-on (2).** A solution of *N*,*N*-dimethylformamide-diethylacetal (28.2 mL, 0.16 mol) in *o*-xylene (20 mL) was added dropwise with vigorously stirring to a solution of 4-acetylpyridine (15 mL, 0.14 mol) in *o*-xylene (30 mL) at room temperature. Then, the reaction mixture was heated under reflux for 4 h. Half the volume of the solvent was evaporated and the residue was dispersed in diethyl ether (30 mL). The product was precipitated by cooling in the refrigerator, filtrated, washed with ice-cooled diethyl ether and dried under vacuum. Yellow solid; yield: 70%; M.p. 105–106 °C; IR (KBr): 1642 (C=O) $cm^{-1}$; $^1$H-NMR ($CDCl_3$, 250 MHz): δ (ppm) = 2.80 (s, 3 H, $CH_3$), 3.02 (s, 3 H, $CH_3$), 5.51 (d, $J$ = 12.25 Hz, 1 H, olefinic H), 7.55 (d, $J$ = 6.00 Hz, 2 H, arom. H), 7.69 (d, $J$ = 12.75 Hz, 1 H, olefinic H), 8.56 (d, $J$ = 6.00 Hz, 2 H, arom. H); $^{13}$C-NMR ($CDCl_3$, 63 MHz): δ (ppm) = 37.05, 44.90, 91.30, 120.81, 146.85, 149.82, 154.82, 186.04.

***N*-(4-methyl-3-nitrophenyl)-4-(4-pyridyl)-2-pyrimidineamine (3).** To a solution of 4-methyl-3-nitrophenylguanidine hydrochloride (1.41 g, 6.1 mmol) in 2-propanol (50 mL) an excess of $K_2CO_3$ (15 mmol) and the corresponding enaminone **2** (0.97 g, 5.5 mmol) were added. The suspension was stirred under reflux for 48 h. The reaction mixture was cooled to room temperature, the precipitated product was filtered off, suspended in water and stirred vigorously at room temperatue. After 1 h of stirring, the product was filtered off, washed with a small portion of ice-cooled 2-propanol and diethylether and dried ($P_4O_{10}$) to yield a first crop of product. A second crop of product was obtained from the first filtrate, which was separated and suspended in water (40 mL). The precipitate was filtered off, washed with a small portion of ice-cooled 2-propanol and diethylether, and dried ($P_4O_{10}$) to yield a second crop of product. Yellow solid; yield: 73%; M.p. 259–262 °C; IR (KBr): 3267 (NH), 1524, 1336 ($NO_2$) $cm^{-1}$; $^1$H-NMR (DMSO-$d_6$, 250 MHz): δ (ppm) = 2.47 (s, 3 H, $CH_3$), 7.42 (d, *J* = 8.50 Hz, 1 H, arom. H), 7.60 (d, *J* = 5.00 Hz, 1 H, arom. H), 7.88 (dd, *J* = 8.50, 2.00 Hz, 1 H, arom. H), 8.10 (d, *J* = 5.50 Hz, 2 H, arom. H), 8.70 (d, *J* = 5.00 Hz, 1 H, arom. H), 8.77 (d, *J* = 5.50 Hz, 2 H, arom. H), 8.79 (d, *J* = 2.00 Hz, 1 H, arom. H), 10.22 (broad s, 1 H, NH); $^{13}$C-NMR (DMSO-$d_6$, 63 MHz): δ (ppm) = 19.43, 109.51, 113.99, 121.08, 123.79, 125.51, 133.07, 139.56, 143.76, 148.78, 150.77, 160.05, 160.29, 161.60.

***N*-(4-methyl-3-aminophenyl)-4-(4-pyridyl)-2-pyrimidineamine (4).** A mixture of phenylamino-pyrimidine **3** (1.0 g, 3.36 mmol) and $PtO_2$ (0.2 g) in THF (50 mL) was vigorously stirred under a static $H_2$ atmosphere at room temperature until no initial compound was detected by TLC. The catalyst was filtered off and the solvent evaporated under vacuum. The residue was treated with diethyl ether and hexane and the product dried under vacuum to give quantitative the pure amino compounds **4**. The corresponding aniline derivative **4**, without further purification, was subsequently coupled with 2-chloro-4-nitrobenzoyl chloride to the final compound **Y18**.

***N*-[2-methyl-5-(4-pyridin-4-yl-pyrimidin-2-ylamino)-phenyl]-2-chloro-4-nitrobenzamide (Y18).** A solution of 2-chloro-4-nitrobenzoyl chloride (0.96 g, 0.44 mmol) in THF (15 mL) was added dropwise to an ice-cold solution of the corresponding amine **4** (0.1 g, 0.37 mmol) and $Et_3N$ (0.50 mmol) in THF (25 mL). The mixture was stirred vigorously at 0 °C for 2 h and at room temperature for 24 h, then poured into ice and extracted with saturated $K_2CO_3$ and $CH_2Cl_2$. The solvent was evaporated under vacuum and the residue precipitated by the addition of diethylether to yield a first crop of product. A second crop of product was obtained from the organic phase, which was separated, washed with water and dried ($Na_2SO_4$). The solvent was evaporated under vacuum and the residue purified by flash chromatography on silica gel (dichloromethane/acetone 5:1). Yellow solid; yield: 87%; M.p. 260–262 °C (dec.); IR (KBr): 3256 (NH), 1655 (C=O), 1528, 1350 ($NO_2$) $cm^{-1}$; $^1$H-NMR (DMSO-$d_6$, 400 MHz): δ (ppm) = 2.29 (s, 3 H, $CH_3$), 7.24 (d, *J* = 8.50 Hz, 1 H, arom. H), 7.55 (d, *J* = 5.50 Hz, 2 H, arom. H), 7.92 (d, *J* = 8.50 Hz, 1 H, arom. H), 8.16 (d, *J* = 5.50 Hz, 2 H, arom. H), 8.21 (s, 1 H, arom. H), 8.35 (dd, *J* = 8.50, 2.00 Hz, 1 H, arom. H), 8.46 (d, *J* = 2.00 Hz, 1 H, arom. H), 8.67 (d, *J* = 5.50 Hz, 1 H, arom. H), 8.76 (d, *J* = 5.50 Hz, 2 H, arom. H), 9.85 (broad s, 1 H, NH), 10.24 (broad s, 1 H, NH); $^{13}$C-NMR (DMSO-$d_6$, 100 MHz): δ (ppm) = 17.95, 108.94, 117.01, 117.87, 121.51, 123.15, 125.21, 126.48, 130.54, 130.89, 131.61, 135.67, 139.00, 143.32, 144.35, 148.85, 151.09, 160.53, 160.81, 161.79, 164.22; HRMS (ESI): calcd for $C_{23}H_{17}ClN_6O_3$ $[M+H]^+$ *m/z* 461.1123, found $[M+H]^+$ *m/z* 461.1113.

**Figure S1.** $^{1}$H-NMR (DMSO-$d_6$, 400 MHz) of final compound-inhibitor **Y18**.

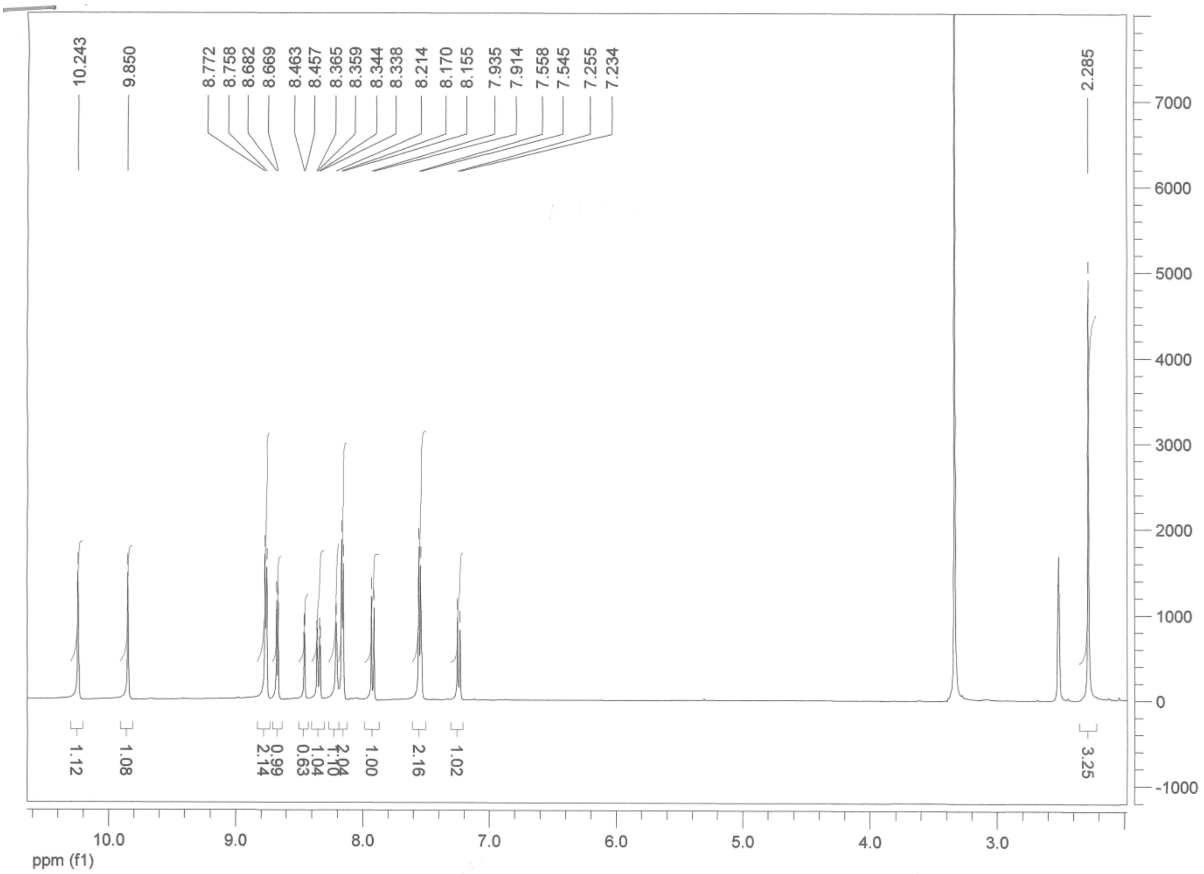


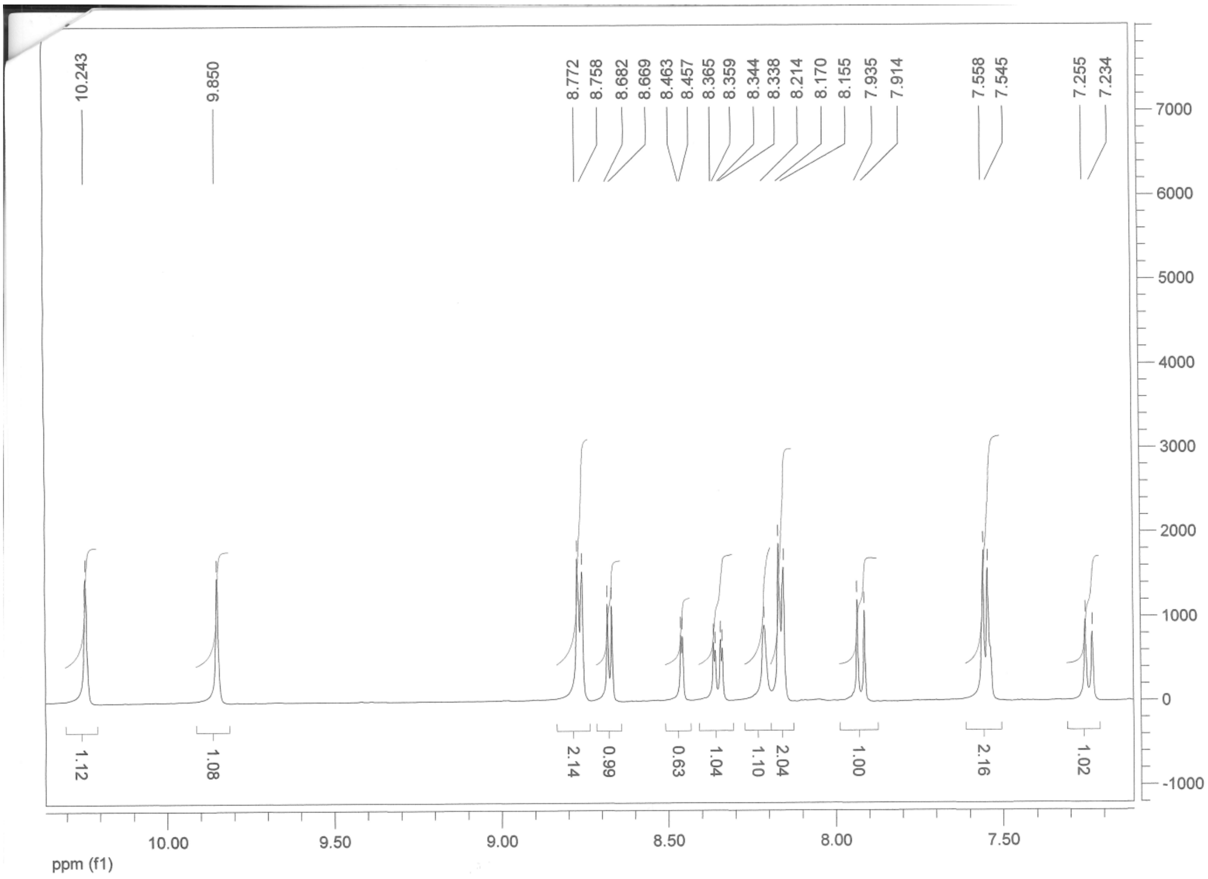

**Figure S2.** $^{13}$C-NMR (DMSO-$d_6$, 100 MHz) of final compound-inhibitor **Y18**.

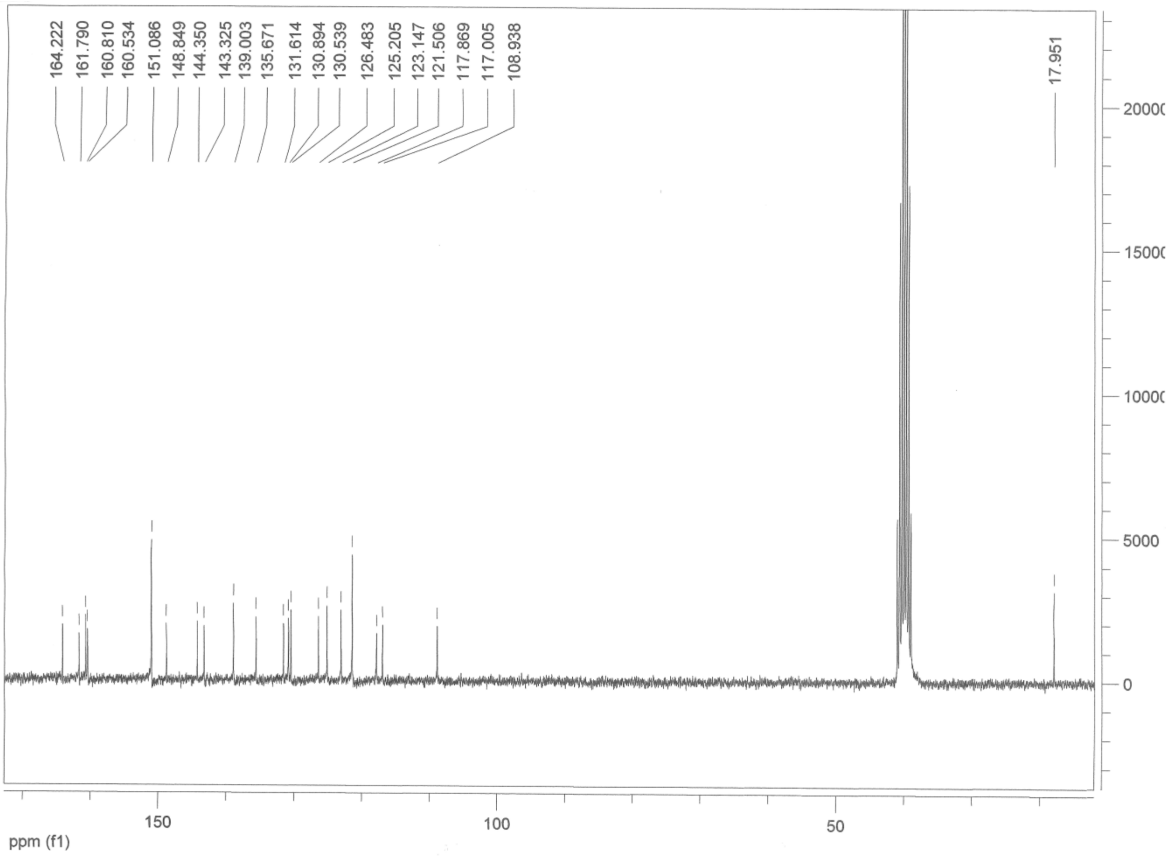


**Figure S3.** HRMS (ESI) of final compound-inhibitor **Y18**.

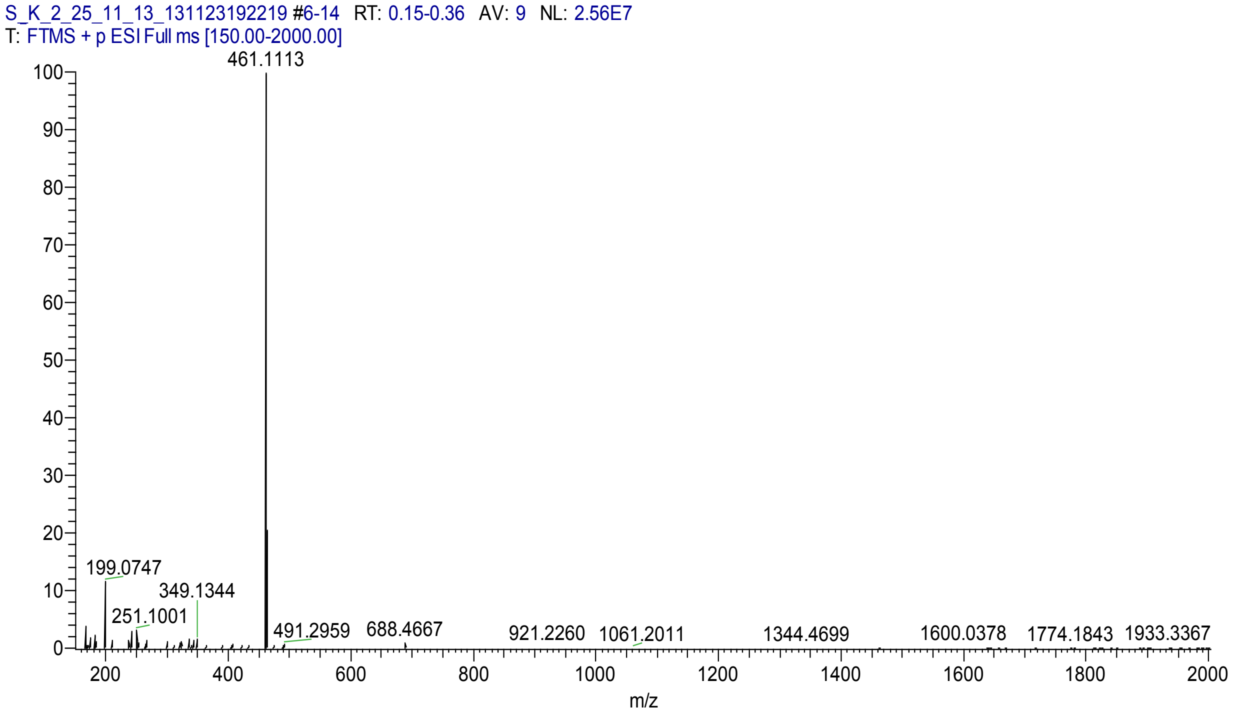

**Figure S3.** *Cont.*

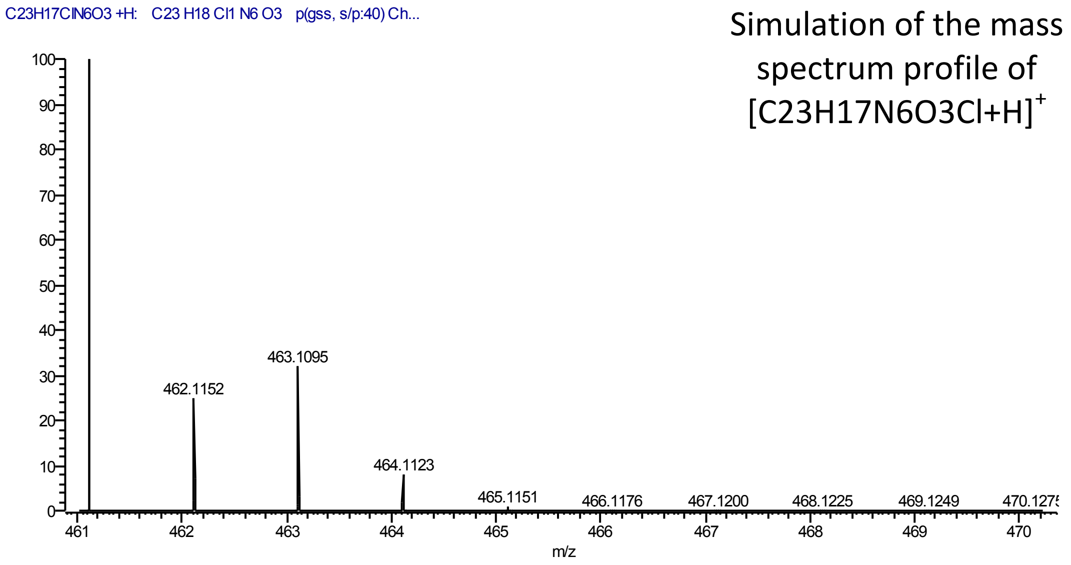


**Elemental composition search on mass 461.1113**
(Isotopes O-16, C-12, H-1, N-14, Cl-35)

| *m/z* | Theo. Mass | Delta (ppm) | RDB equiv. | Composition |
|---|---|---|---|---|
| 461.1113 | 461.1123 - | 2.35 | 17.5 | C23 H18 O3 N6 Cl |

## Cell Cultures

K562 cells were cultured in RPMI medium which was enriched with 10% fetal bovine serum and 1% mixture of penicillin/streptomycin (1:1). The cells were incubated at 37 °C with 5% $CO_2$ in a humidified atmosphere and medium renewal every 2–3 days. The Y18 compound was dissolved in DMSO (concentration 10 mM—Stock Solution).

K562 cells ($5 \times 10^3$) were treated with various concentrations (Table 1) of the Y18 compound in 96-well plates for 48 h (37 °C, 5% $CO_2$ in a humidified atmosphere). After 48 h of incubation, the cell viability was determined by adding of MTT (3-(4,5-Dimethyl-2-thiazolyl)-2,5-diphenyl-2H-tetrazolium bromide) solution in each well. The plate was incubated for another 4 h (37 °C, 5% $CO_2$ in a humidified atmosphere). After remove of the medium, the formazan crystals formed in the wells were dissolved in DMSO and measurements of the absorbance were made at 540 nm. The $IC_{50}$ 1.13 ± 0.15 μM was estimated for the Y18 compound.

## References

1. Bredereck, H.; Effenberger, F.; Bosch, H. Säureamid-Reaktionen, XLV. Untersuchungen über die Reaktionsfähigkeit von Formamidinen, Dimethylformamid-diäthylacetal (Amidacetal) und Bis-dimethyl-amino-methoxy-methan (Aminalester) *Ber. Dtsch. Chem. Ges.* **1964**, *97*, 3397–3406.

2. Zimmermann, J.; Buchdunger, E.; Mett, H.; Meyer, T.; Lydon, N.B.; Traxler, P. Phenylamino-pyrimidine (PAP)—derivatives: A new class of potent and highly selective PDGF-receptor autophosphorylation inhibitors *Bioorg. Med. Chem. Lett.* **1996**, *6*, 1221–1226
3. Zimmerman, J.; Buchdunger, E.; Mett, H.; Meyer, T.; Lydon, N.B. Potent and selective inhibitors of the Abl-kinase: Phenylamino-pyrimidine (PAP) derivatives *Biorg. Med. Chem. Lett.* **1997**, *7*, 187–192
4. Skobridis, K.; Kinigopoulou, M.; Theodorou, V.; Giannousi, E.; Russell, A.; Chauhan, R.; Sala, R.; Brownlow, N.; Kiriakidis, S.; Domin, J.; *et al.* Novel imatinib derivatives with altered specificity between Bcr-Abl and FMS, KIT, and PDGF receptors. *ChemMedChem* **2010**, *5*, 130–139.

© 2014 by the authors; licensee MDPI, Basel, Switzerland. This article is an open access article distributed under the terms and conditions of the Creative Commons Attribution license (http://creativecommons.org/licenses/by/3.0/).
